# Successive ionic layer adsorption and reaction (SILAR) -driven cobalt oxide integration on pencil graphite for efficient electrochemical oxygen evolution reaction in alkaline medium

**DOI:** 10.1039/d6ra04902h

**Published:** 2026-07-29

**Authors:** Mahfuz Rana, Kazi Hamidur Rashid, Abrar Yasir Abir, Faiza Jan Iftikhar, Motasim Bin Islam, Md. Mahmudul Hasan, Mohammad Anwar Parvez, Mostafizur Rahaman, Syed Kashif Ali, Mohammad A. Hasnat

**Affiliations:** a Department of Chemistry, Pabna University of Science and Technology Pabna-6600 Bangladesh; b Electrochemistry & Catalysis Research Laboratory (ECRL), Department of Chemistry, School of Physical Sciences, Shahjalal University of Science and Technology Sylhet-3114 Bangladesh mah-che@sust.edu; c NUTECH School of Applied Sciences & Humanities, National University of Technology Islamabad 44000 Pakistan; d Research Organization for Nano & Life Innovation, Waseda University Tokyo 162-0041 Japan; e Department of Chemical Engineering, College of Engineering, King Faisal University P.O. Box 380 Al-Ahsa 31982 Saudi Arabia; f Department of Chemistry, College of Science, King Saud University P.O. Box 2455 Riyadh 11451 Saudi Arabia; g Department of Physical Sciences, Chemistry Division, College of Science, Jazan University P.O. Box. 114 Jazan 45142 Kingdom of Saudi Arabia

## Abstract

Sustainable energy conversion depends on the development of effective and economical electrocatalysts. In this work, we highlight the development of cobalt oxide (Co_3_O_4_) as an electrocatalyst by employing a scalable and economical Successive Ionic Layer Adsorption and Reaction (SILAR) method onto an electrically activated pencil graphite (Ac-PGE) as an affordable substrate for monitoring the oxygen evolution reaction (OER). According to electrochemical impedance spectroscopy, the SILAR process produced uniform deposition and improved surface activation, which resulted in a considerably reduced charge transfer resistance (*R*_ct_) of 0.08 kΩ. The OER overpotential was observed at 240 mV at 10 mA cm^−2^ with a Tafel slope of 47.57 mV dec^−1^, and a turnover frequency of 0.082 s^−1^ at the activated electrode. LSV and OCP demonstrate that Co_3_O_4_@Ac-PGE performs better electrochemically than the other electrodes under investigation (In-PGE, Ac-PGE, and Co_3_O_4_@In-PGE). Additionally, after 8 hours, it maintained more than 93% of its initial activity, demonstrating exceptional endurance. Overall, it was observed that the Co_3_O_4_@Ac-PGE electrode developed by the SILAR method outperforms a number of traditional and noble-metal-based catalysts and offers a practical, long-lasting, and financially sustainable approach to effective water-splitting and renewable energy conversion. The structural and surface properties of the modified electrodes were investigated using energy-dispersive X-ray spectroscopy (EDX), field emission scanning electron microscopy (FESEM), and X-ray photoelectron spectroscopy (XPS). This work shows a scalable and cost-effective strategy to design an efficient electrocatalyst by using SILAR for OER, which can contribute towards Green Hydrogen production.

## Abstract 2

Sustainable energy conversion depends on the development of effective and economical electrocatalysts.

## Graphical abstract

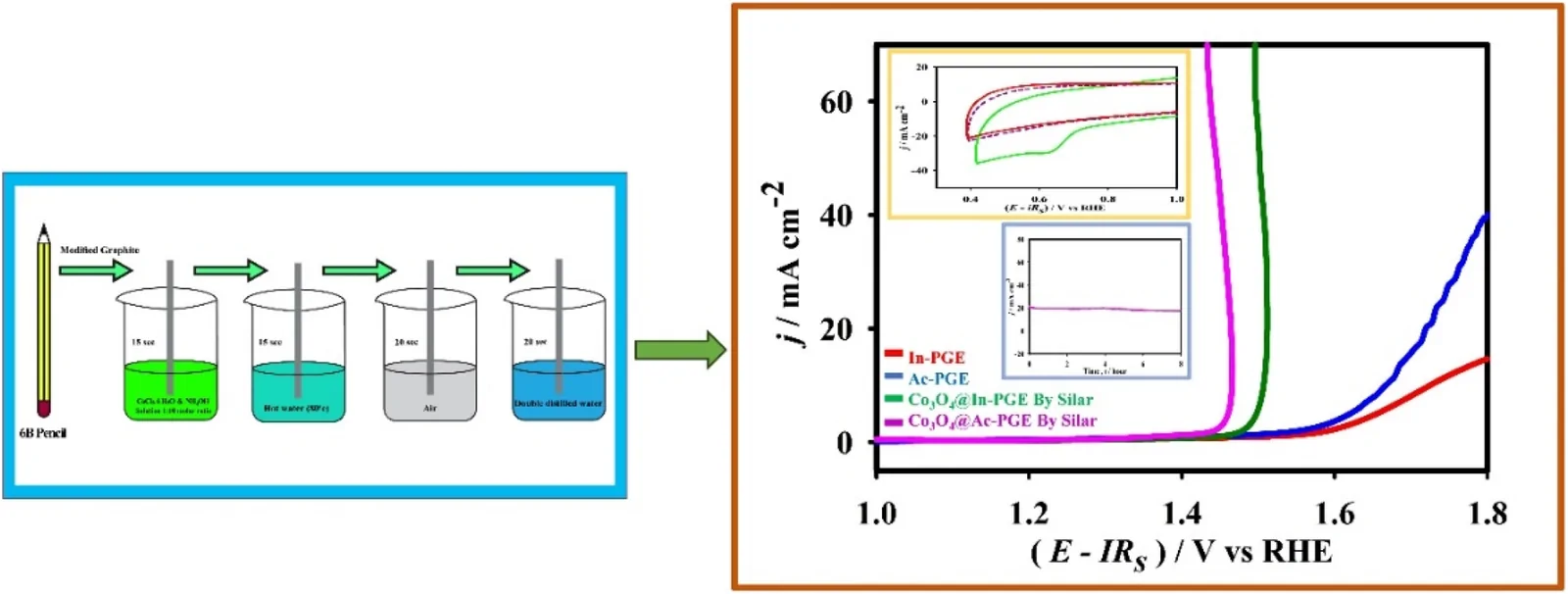

## 1. Introduction

Extensive dependence on non-renewable fossil fuels has largely been driven by an exponential population rise, concurrent economic expansion, and boom in industrialization. However, this dependence has caused substantial environmental damage, exacerbating climate change, and escalating energy inequity. As a result, global warming has intensified over the decades and consequently the ecological equilibrium has become upset by the rise in greenhouse gas emissions (GHGs).^1–3^ Long-term fossil fuel use not only increases air pollution but also jeopardizes energy security and sustainable development.^4^ In light of this, the advancement of clean and renewable energy technology has emerged as a global priority.^5^ Hence, extensive research is focused on developing and exploring energy conversion and storage systems that are inexpensive, highly effective, and environmentally sustainable, such as solar,^6^ wind^7^ and electrochemical technologies.^8^ Among these, electrochemical techniques present a particularly promising strategy since they allow for effective energy conversion and storage through processes like fuel production and water splitting.^9^ In order to reduce carbon emissions and to achieve a sustainable energy future, these cutting-edge solutions are anticipated to be crucial.^10^

The oxygen evolution reaction (OER) is a crucial reaction in different electrochemical energy storage and transformation systems, for instance regenerative fuel cells,^11^ rechargeable metal-air batteries^12^ and water electrolysis.^13^ Yet to generate clean energy at a reasonable cost, electrochemical water splitting which can be carried out in different media such as neutral, alkaline, and acidic is widely utilized.^14–17^ Intrinsically, the OER process occurs following a four-electron transfer pathway that results in sluggish kinetics with high overpotential and substantial energy loss^18,19^ which is an important indicator of the system's overall efficiency.^20^ So, the manufacturing of stable, inexpensive, and active OER electrocatalysts has been a holy grail in this domain of study.^21–23^ Noble metal-based electrocatalysts such as RuO_2_ and IrO_2_ have strong catalytic efficiency in both acidic and alkaline conditions, and are considered benchmark catalysts for the OER process.^24–26^

For instance, Rajashekar Badam *et al.* reported hydrothermal production of evenly supported IrO_2_ nanoparticles (∼1.7 nm) on carbon nanotubes (CNTs) functionalized with –COOH and tested their electrochemical performance in acidic medium with outstanding results.^27^ However, the high-cost and limited geographical supply of these noble metals, as well as their poor stability in operation due to dissolution or conversion of their reduced forms, severely restrict their large-scale utilization.^28–30^ Therefore, researchers are gradually shifting away from noble metal catalysts and turning toward more affordable and practical transition-metal based electrocatalysts for achieving improved activity for OER.^31–33^ Previous studies have demonstrated the potential of Co_3_O_4_ based electrocatalysts for OER. However, critical limitations still hinder their practical application.^34^ For example, Jakub A. Koza *et al.* have reported Co_3_O_4_ crystalline films deposited on stainless steel *via* electrodeposition for OER activity. The electrocatalyst exhibited a few limitations such as complex synthesis, sensitivity to environmental conditions, and poor adhesion to substrate and mechanical instability, restricting its scalability.^35^ Similarly, Yang *et al.* documented Co_3_O_4_ nanosheets grown on carbon substrates that showed improved catalytic activity for OER. Nevertheless, their synthesis relied on a multistep approach with controlled environment, and the stability was highly dependent on substrate adhesion, with limited mechanistic understanding.^36^ Another approach was to integrate Co_3_O_4_ with conductive carbon substrates such as carbon cloth and graphene that enhance charge transfer and increase active surface area, still these are not without drawbacks.^37^ T. V. M. Sreekanth *et al.* electrodeposited Co_3_O_4_ on carbon cloth that demonstrated reasonable activity and durability but relied on strict deposition conditions and post treatment processes.^38^ Likewise, Qian-Yu Wang *et al.* observed that graphene supported Co_3_O_4_ significantly outperforms commercial Co_3_O_4;_ however, the use of template assisted or multi-step fabrication strategies increased synthetic complexity and limit scalability.^39^

These limitations highlight that the performance of Co_3_O_4_/carbon electrodes is strongly governed by the deposition strategy, and existing methods fail to simultaneously achieve simplicity, scalability, and performance. Therefore, the development of a straightforward and controllable approach is essential for efficient OER applications.^40^ Among the available deposition techniques, the Successive Ionic Layer Adsorption and Reaction (SILAR) method was selected for the fabrication of Co_3_O_4_ films due to its simplicity, scalability, rapid and controllable film growth, uniform deposition, strong substrate adhesion under mild operating conditions, binder-free film formation, and its relatively eco-friendly and cost-effective processing route.^41^ Thus, the combined strategy of electrochemically-activated graphite and SILAR deposition provides an efficient and scalable route to overcome the limitations of previously reported methods for high-performance OER electrocatalysts. These attributes make it a promising and scalable strategy for the development of efficient Co_3_O_4_ -based OER electrocatalysts.^42,43^

In this research, we have developed a Co_3_O_4_ modified pencil graphite electrode applying the SILAR method in alkaline medium, which has not been previously employed for OER to the best of our knowledge. Here, pencil graphite was tested in four different configurations, such as, inactive (pristine) pencil graphite (In-PGE), activated pencil graphite (Ac-PGE), Co_3_O_4_ deposited on inactive pencil graphite by SILAR method (Co_3_O_4_@In-PGE), Co_3_O_4_ deposited on activated pencil graphite by SILAR method (Co_3_O_4_@Ac-PGE by SILAR), and the best configuration was then analyzed further. Open circuit potential (OCP), electrochemical impedance spectroscopy (EIS), cyclic voltammetry (CV), chronoamperometry (CA), and linear sweep voltammetry (LSV) were applied to evaluate the OER performance using the developed electrodes. In essence, the work effectively outlined a scalable path towards the development of an extremely effective OER electrocatalyst made up of non-precious metals by using an economical and scalable SILAR method.

## 2. Experimental

### 2.1 Chemicals, reagents & instruments

All chemicals employed in this experiment were of analytical grade and had maximum purity. Cobalt chloride hexahydrate (CoCl_2_·6H_2_O, ≥98%), sodium hydroxide (NaOH, ≥98%) and ammonium hydroxide (NH_4_OH, 28–30 wt%) were purchased from Sigma Aldrich (St. Louis, MO, USA). Ultrapure Milli-Q water was used in the formulation of all requisite solutions to ensure exclusion of impurities that could compromise the results.

For electrochemical investigations, a CHI-660 potentiostat (CHI Instruments, USA), PINE Wave driver 20 (Pine Research, USA), and Autolab Potentiostat (PGSTAT128N, Netherlands) in a standard three electrode configuration were used. A three-electrode Pyrex glass cell, an N_2_ cylinder, and a pH meter were also used for the experiments. The working electrodes were In-PGE, Ac-PGE, Co_3_O_4_@In-PGE, Co_3_O_4_@Ac-PGE with a geometrical area of 0.0707 cm^2^, where Pt wire and Ag/AgCl (sat. KCl) were respectively employed as counter and reference electrodes.

The structural and surface morphological characteristics of the synthesized materials were investigated using different analytical techniques. Surface morphology was examined by FESEM, whereas elemental composition was determined through EDX analysis using a TM3030Plus miniscope (Hitachi Ltd). XPS measurements were conducted using a Kratos Axis Ultra DLD spectrometer equipped with an Al Kα X-ray source (1486.6 eV).

### 2.2 Electrode fabrication

**Scheme 1:**
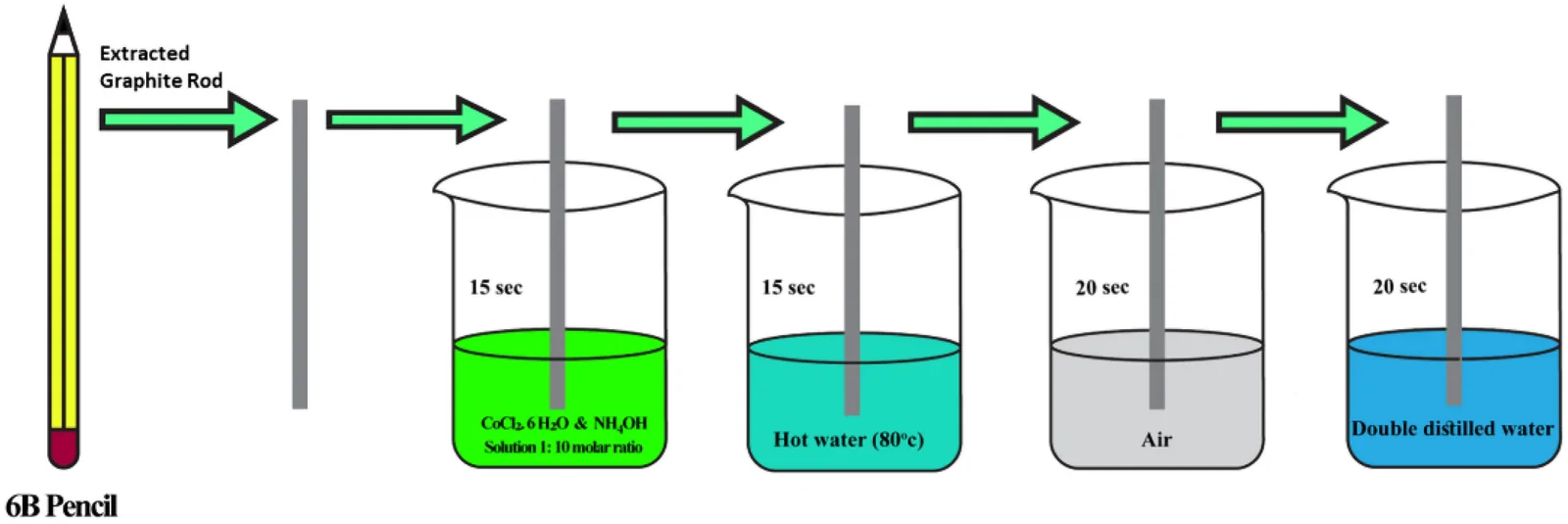
Electrode preparation diagram using SILAR method.

For electrode fabrication, we collected graphite rod from a 6B pencil, which had a diameter of 3 mm. The outside of the rod was then covered with epoxy resin leaving only the active geometric area. In the first instance, two of these pencil graphite electrodes, which are referred to as Ac-PGE were electrochemically activated by cycle voltammetry within the potential window of +0.952 to +2.752 V *vs.* RHE with 100 mV s^−1^ scan rate for 20 cycles, where 1 M NaOH solution was used as an electrolyte. In the second instance, two other PGEs, referred to as In-PGE, were not activated. Out of these, one Ac-PGE and one In-PGE surfaces were modified with Co_3_O_4_ thin film following SILAR method which involves a series of steps to optimize the surface properties of the electrode.^44^ The initial step in the modification procedure was to submerge an In-PGE/Ac-PGE in a CoCl_2_·6H_2_O/NH_4_OH complex solution with a molar ratio of 1 : 10 for 15 seconds. After that, the In-PGE/Ac-PGE was submerged in hot water at 80 °C for an additional 15 seconds, which helped to produce a consistent cobalt oxide deposition on the graphite surface. To get rid of extra water, the electrode was then allowed to dry in the air for 20 seconds. The electrode was then placed in room temperature water for 20 seconds and then was dried in air for another 20 seconds.^41^Scheme 1 summarizes the electrode fabrication process.

### 2.3 Electrochemical measurements

The OER experiments were carried out in a conventional three-electrode setup as given in section 2.1. Several techniques were systematically employed to assess the electrocatalytic performance and stability of the catalysts. Chronoamperometry (CA) was utilized to assess long-term operational durability under a constant applied potential, while OCP measurements were conducted to determine the equilibrium potential. EIS was performed to elucidate the *R*_ct_ at the electrode–electrolyte interface. Additionally, CV and LSV were used to explore the overpotential and overall OER efficiency. All data were processed and interpreted using SigmaPlot (version 10) software. All measured potentials were altered to the reversible hydrogen electrode (RHE) scale, as the original measurements were conducted using an Ag/AgCl (saturated KCl) reference electrode, according to eqn (1).^45^

1 *E*_RHE_ = *E*_Ag/AgCl_ + 0.059pH + 0.197

## 3. Results and discussion

### 3.1 Material characterization

**Fig. 1:**
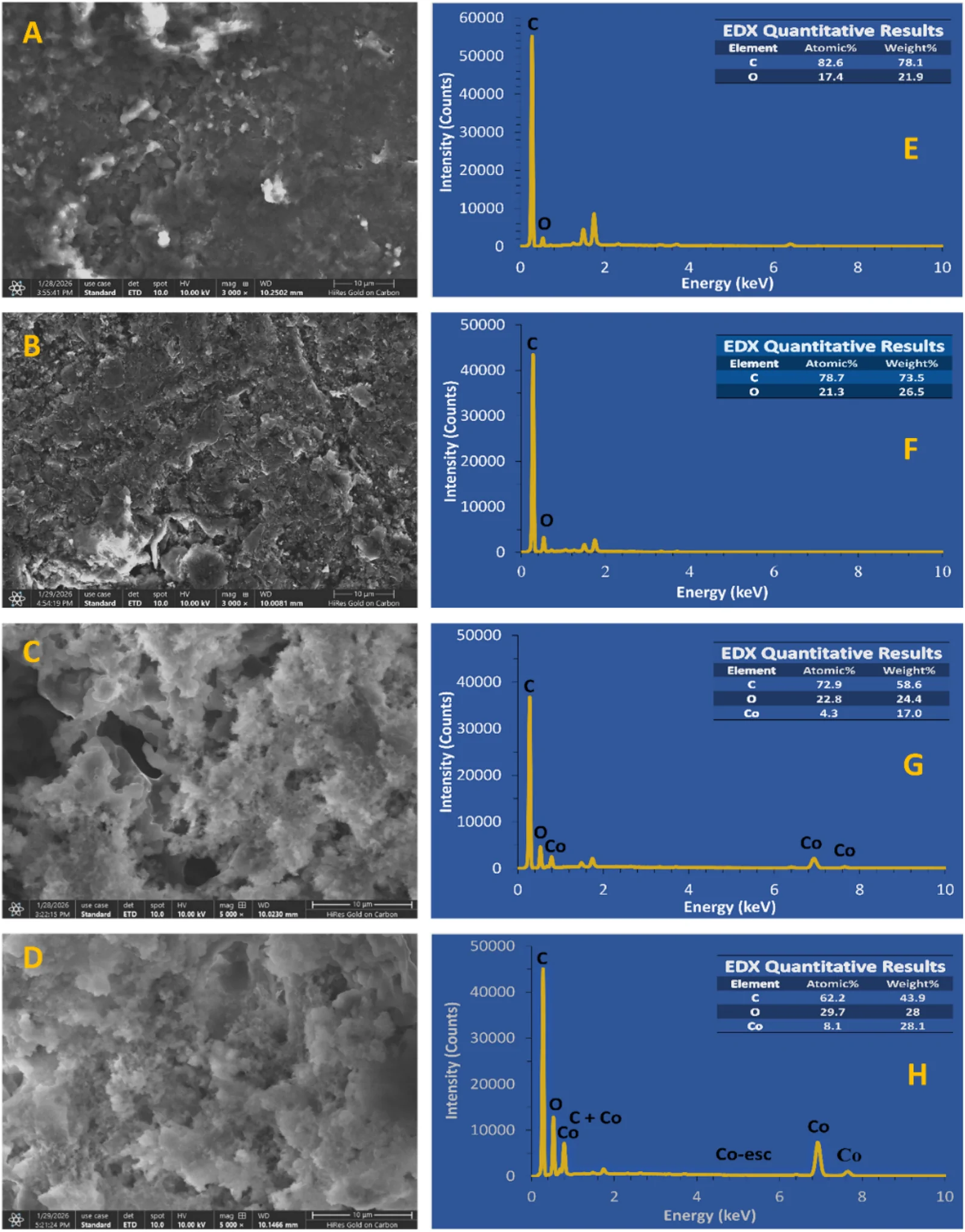
SEM and EDX of In-PGE (A and E), Ac-PGE (B and F), Co_3_O_4_@In-PGE (C and G), and Co_3_O_4_@Ac-PGE (D and H). Activation was performed in 1 M NaOH using CV from +0.952 to +2.752 V *vs.* RHE at 100 mV s^−1^, confirming surface modification and Co_3_O_4_ deposition.

The surface morphology of the prepared electrodes was examined by SEM at a magnification of 10 µm, as presented in Fig. 1(A–D). The SEM image of the inactive pencil graphite electrode (In-PGE, Fig. 1A) shows a relatively smooth and layered graphite surface. After electrochemical activation in 1 M NaOH using CV within a potential range of +0.952 to +2.752 V *vs.* RHE at a scan rate of 100 mV s^−1^, the activated electrode (Ac-PGE, Fig. 1B) exhibits a rougher and more defect-rich morphology, indicating formation of additional electroactive sites.^46,47^ Following Co_3_O_4_ deposition *via* SILAR method, the Co_3_O_4_@In-PGE electrode (Fig. 1C) displays aggregated and porous nanostructures distributed over the graphite surface, confirming successful material growth. In contrast, the Co_3_O_4_@Ac-PGE electrode (Fig. 1D) shows a more uniform and densely packed porous structure, suggesting that the activated surface promotes better nucleation and loading of Co_3_O_4_.^48,49^ To further verify the elemental composition, EDX analysis was performed (Fig. 1(E–H)). The spectra confirmed the presence of carbon and oxygen in the graphite electrodes, while additional characteristic peaks of cobalt demonstrate the successful incorporation of Co_3_O_4_ in the modified sample electrodes. Notably, the stronger cobalt signals observed in Co_3_O_4_@Ac-PGE indicate enhanced material deposition, expected to promote the electrocatalytic activity of the electrode.^50^

**Fig. 2:**
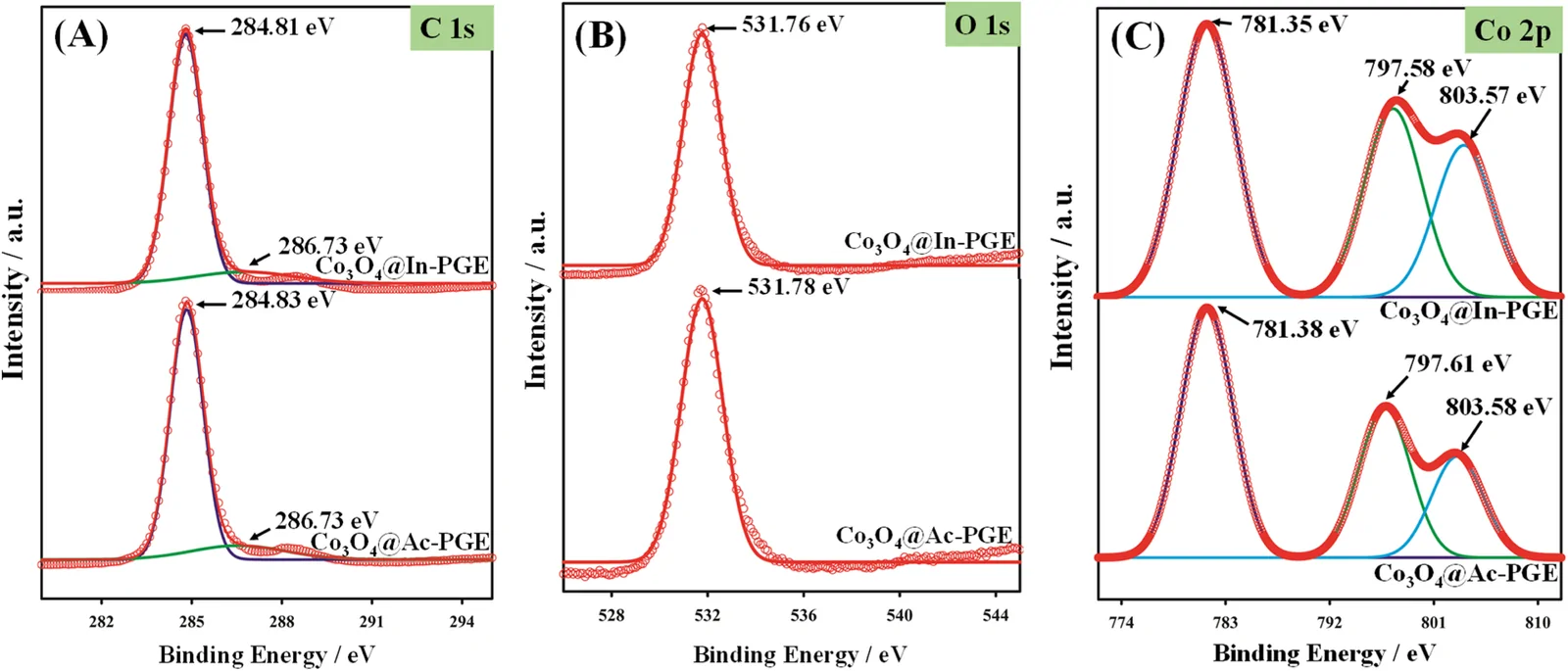
High-resolution XPS spectra of In-PGE and Ac-PGE after Co_3_O_4_ deposition *via* SILAR method for, (A) C 1s, (B) O 1s, and (C) Co 2p regions.

To investigate the surface states and electronic structure of the modified electrodes, X-ray photoelectron spectroscopy (XPS) was performed for Co_3_O_4_@In-PGE and Co_3_O_4_@Ac-PGE after SILAR deposition. Comparative XPS spectra in the C 1s, O 1s and Co 2p regions are illustrated in Fig. 2. The C 1s spectra of both active and inactive electrodes are dominated by the sp^2^ carbon peak at ∼284.8 eV, which is for the graphite framework.^51^ Comparing with Co_3_O_4_@In-PGE, Co_3_O_4_@Ac-PGE shows a slight positive shift to 284.83 eV. This positive shift indicates a small decrease in electron density due to electrochemical activation and the introduction of oxygen-containing functional groups.^52^ A second component at 286.73 eV, assigned to –C=O species, relatively intensely appeared for Ac-PGE due to oxidation of carbon atoms on the graphite surface. The overall observation confirms that activation mainly increases the abundance of the oxygen functionalities.^52,53^ The O 1s spectra further reveal differences in surface oxygen chemistry. The main O 1s peak shifts slightly from 531.76 eV for Co_3_O_4_@In-PGE to 531.78 eV for Co_3_O_4_@Ac-PGE. This binding energy range is commonly attributed to surface hydroxyl groups, and defect-related oxygen rather than lattice oxygen in crystalline Co_3_O_4_.^54,55^ The small positive shift suggests a slightly more electron-deficient oxygen environment resulting from stronger interfacial interactions between Co_3_O_4_ and the oxygen-functionalized graphite substrate.^56^ Moreover, the sharper and more intense O 1s peak of Co_3_O_4_@Ac-PGE indicates a higher concentration of oxygen species on the surface, particularly hydroxyl groups, which facilitate the adsorption and conversion of OER intermediates during the adsorbate evolution mechanism.^57,58^

The Co 2p spectra exhibit the characteristic Co 2p_3/2_ and Co 2p_1/2_ doublet together with shake-up satellite peaks. These peaks confirm the successful deposition of cobalt oxide on both electrodes. In Co_3_O_4_@In-PGE, the Co 2p_3/2_ and Co 2p_1/2_ peaks are located at 781.35 and 797.58, respectively, while in Co_3_O_4_@Ac-PGE they shift slightly to 781.38 and 797.61 eV. The corresponding shake-up satellite peaks appear at 803.57 and 803.58 eV, respectively.^59,60^ The spin–orbit splitting remains constant at 16.23 eV, confirming the reliability of the spectral fitting and indicating that the observed shifts arise from changes in the local electronic environment rather than changes in cobalt oxidation state.^58,61^ The binding energies and prominent satellite features are characteristic of mixed-valence spinel Co_3_O_4_ containing both Co^2+^ and Co^3+^ species. The slight positive shift in Co 2p binding energy is attributed to electron withdrawal by the oxygen-functionalized graphite support, which indicates the Co–O–C interfacial interaction and slightly reduces the electron density around cobalt atoms.^62,63^ Such enhanced electronic coupling can improve charge transfer, stabilize active cobalt species under anodic conditions, and optimize the adsorption of OER intermediates, thereby contributing to the superior OER activity of Co_3_O_4_@Ac-PGE.^64,65^

### 3.2 Open circuit potential (OCP) studies

The OCP is the working electrode's potential measured in relation to the reference electrode when there is no current passing through a three-electrode system.^66^ The OCP is caused by the separation of charges at the electrode–electrolyte interface. This potential controls the electrode's propensity to get reduced or oxidized.^67^ For this experiment, the OCP measurements were performed for In-PGE, Ac-PGE, Co_3_O_4_@In-PGE, and Co_3_O_4_@Ac-PGE electrodes in 1.0 M NaOH to elucidate the electrochemical characteristics arising from electrode–electrolyte interfacial interactions.

According to Fig. S1 and S6, the OCP values for the In-PGE, Ac-PGE, Co_3_O_4_@In-PGE, and Co_3_O_4_@Ac-PGE electrodes were, +0.83 V, +0.90 V, +1.01 V, and +1.11 V *vs.* RHE, respectively. Here, it is evident that Co_3_O_4_ deposited using the SILAR method on In-PGE and Ac-PGE exhibited a higher positive OCP value. More positive OCP values indicate a greater propensity for oxidation and better charge transfer, both of which contribute towards increased OER activity.^68^ Co_3_O_4_@Ac-PGE has the maximum OCP value of all four electrodes in this instance, making it the most effective for displaying OER activity.

### 3.3 Electrochemical impedance spectroscopy

**Fig. 3:**
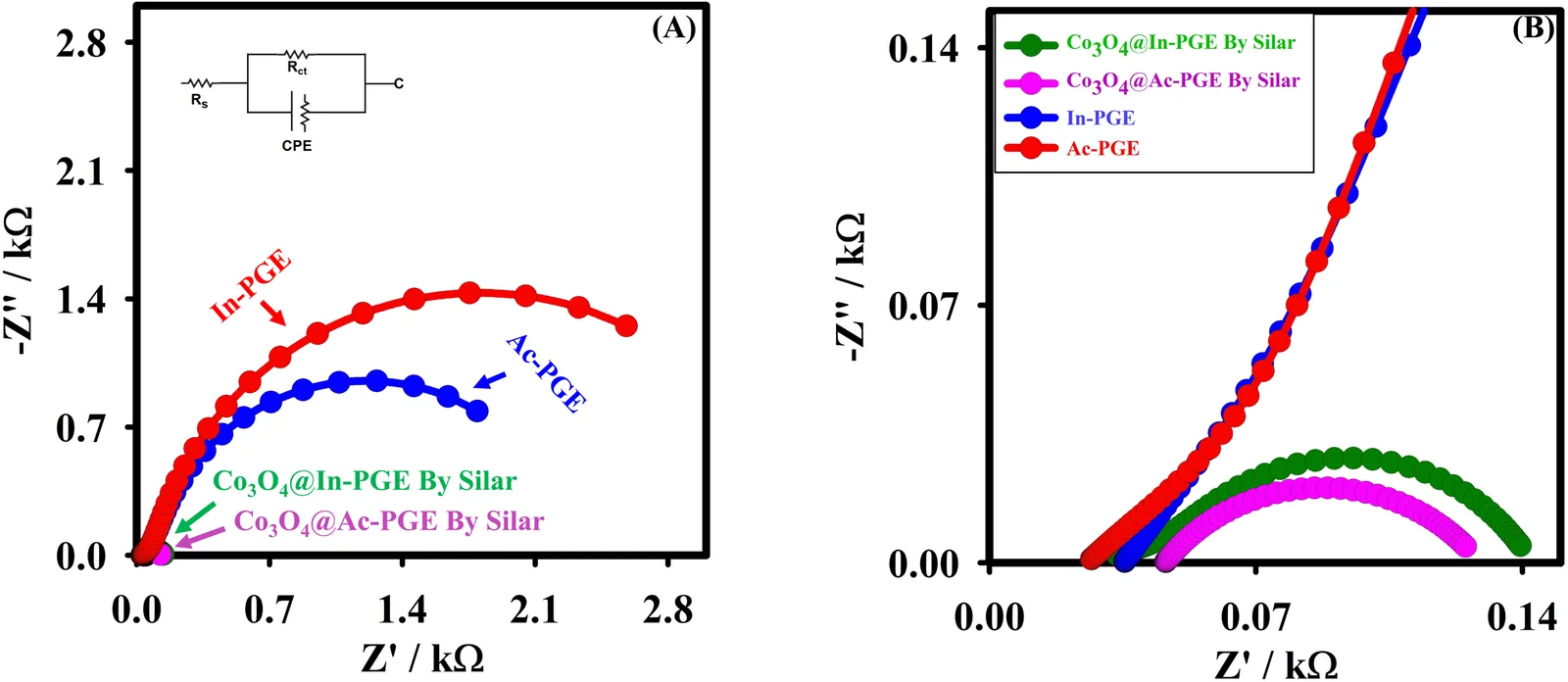
(A) Nyquist plots obtained at an excitation potential of 1.55 V *vs.* RHE for In-PGE, Ac-PGE, Co_3_O_4_@In-PGE, Co_3_O_4_@Ac-PGE electrodes in 1.0 M NaOH electrolyte. (B) Magnified view shows semi-circular behavior for the different modified electrodes in the high frequency region.

EIS is a potent method for examining the charge transfer mechanisms and interfacial characteristics at the electrode–electrolyte interface. To investigate the impedance response of the system, a small amplitude AC potential is applied across a variety of frequencies. In order to comprehend the electrochemical performance of electrode materials, this approach delivers essential metrics like solution resistance (*R*_s_), charge transfer resistance (*R*_ct_) and and double-layer capacitance (*C*_dl_).^69^ In this study, the *R*_ct_ of four different as-modified electrodes were investigated using EIS measurements. The experiments were conducted in an electrolyte of 1.0 M NaOH at an applied voltage of 1.55 V *vs.* RHE. Fig. 3 demonstrates the charge transfer kinetics primarily driving the electrochemical processes. To compare with unmodified electrodes, the Co_3_O_4_ modified electrodes made using the SILAR approach showed the smallest semicircle widths, indicating noticeably lower *R*_ct_ values.

As shown in Fig. 3 and summarized in Table S1, the In-PGE electrode exhibited the largest semicircle, corresponding to an *R*_ct_ of 3.50 kΩ, which reflects sluggish electron transfer kinetics. In contrast, the Co_3_O_4_@Ac-PGE electrode demonstrated a markedly smaller semicircle with an *R*_ct_ of only 0.08 kΩ, indicating superior charge transfer efficiency. The Ac-PGE and Co_3_O_4_@In-PGE electrodes displayed intermediate *R*_ct_ values of 2.29 kΩ and 0.12 kΩ, respectively. The remarkably low *R*_ct_ value of the Co_3_O_4_@Ac-PGE electrode can be attributed to the homogeneous and uniform deposition of a thin Co_3_O_4_ layer on the activated PGE surface. This coating enhances the amount of electrochemically active sites, facilitates faster charge transport, and minimizes interfacial resistance. These findings clearly demonstrate improved electron transfer kinetics and enhanced catalytic performance of Co_3_O_4_@Ac-PGE compared to other electrodes.^70^ Overall, the EIS results confirm that the Co_3_O_4_@Ac-PGE electrode demonstrates the lowest *R*_ct_ value and superior electrical conductivity among all the tested samples, positioning it as an efficient electrocatalyst with enhanced charge transfer kinetics.

### 3.4 OER performance

OER is a four-electron transfer process and the following mechanism (eqn (2)–(5)) is an accepted pathway taking place at the electrode surface.^71^

2 Catalyst + OH^−^ → catalyst–OH_ads_ + e^−^

3 Catalyst − OH_ads_ + OH^−^ → catalyst–O_ads_ + H_2_O

4 Catalyst − O_ads_ + OH^−^ → catalyst–OOH_ads_ + e^−^

5 Catalyst − OOH_ads_ + OH^−^ → catalyst + H_2_O +O_2_

**Fig. 4:**
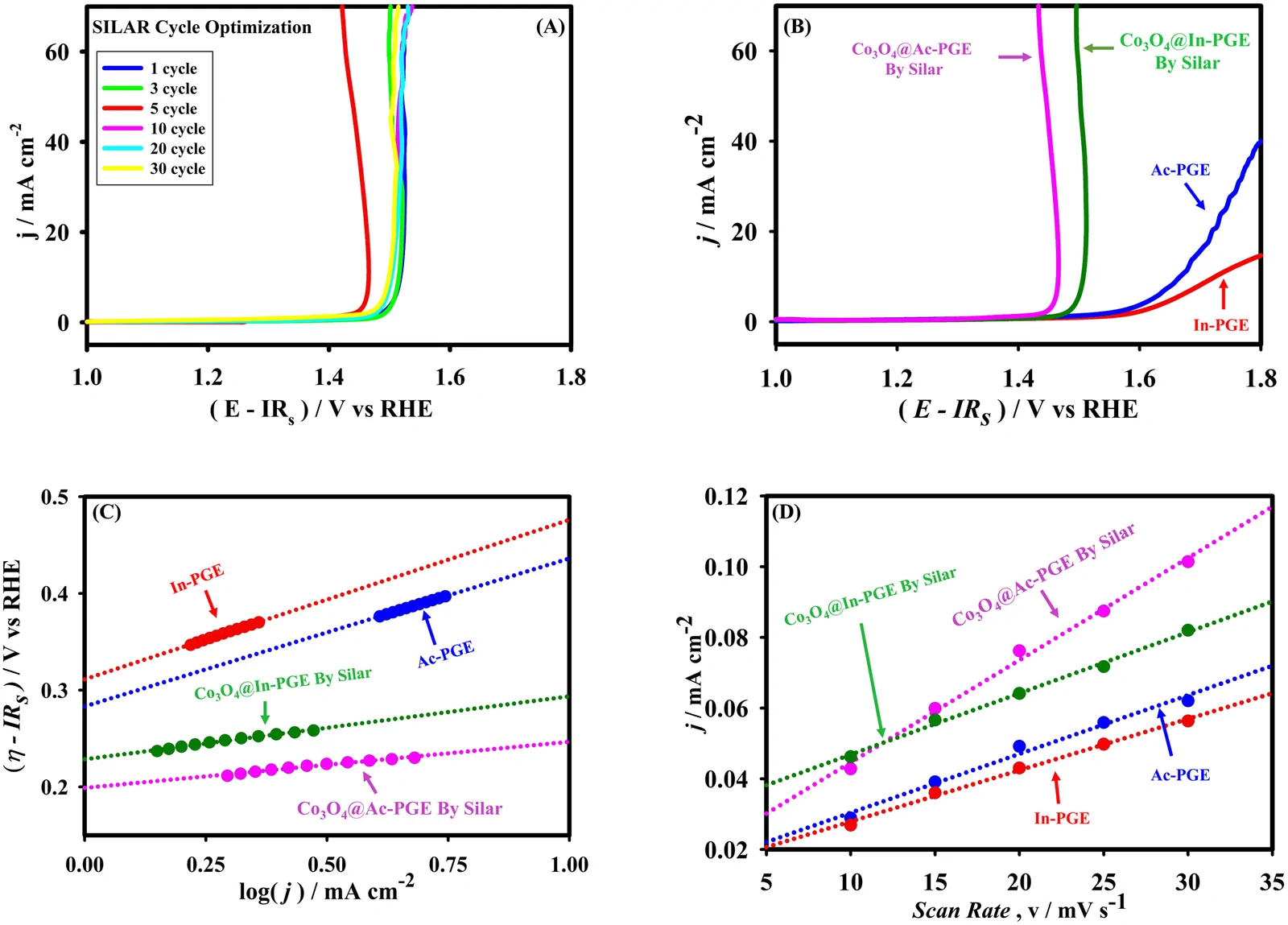
(A–D) Electrochemical performance assessment of the fabricated electrodes toward the oxygen evolution reaction (OER) in 1.0 M NaOH. (A) Optimization of Co_3_O_4_ deposition through variation of SILAR cycles (1–30). (B) Comparative LSV curves of In-PGE, Ac-PGE, Co_3_O_4_@In-PGE, and Co_3_O_4_@Ac-PGE electrodes recorded at a scan rate of 10 mV s^−1^ (C) corresponding OER Tafel plots, and (D) linear plots of current density *versus* scan rate for the investigated electrodes.

To evaluate the OER activity, linear sweep voltammetry (LSV) measurements were performed by sweeping the potential from 0.75 to 1.95 V *vs.* RHE at a scan rate of 10 mV s^−1^. Prior to the comparative electrochemical investigation, the SILAR deposition conditions were optimized by varying the number of deposition cycles from 1 to 30. The corresponding polarization curves obtained for the electrodes prepared with different SILAR cycles are presented in Fig. 4(A), while the associated onset potentials, overpotentials, and Tafel slope values are summarized in Table S2 (SI). Among the investigated conditions, the electrode fabricated with 5 SILAR cycles delivered the most favorable overall OER performance. The superior activity is attributed to the formation of an optimally deposited Co_3_O_4_ layer that maximizes the utilization of electrochemically active sites while maintaining efficient charge-transfer pathways.^72,73^ At lower cycle numbers, incomplete surface coverage may limit the density of accessible active sites, whereas excessive deposition can result in a thicker catalyst layer that imposes additional resistance to charge transport and mass diffusion.^72,74^ Consequently, 5 SILAR cycles were identified as the optimum deposition condition and employed for all subsequent electrochemical studies. The OER activities of the optimized electrode and the control electrodes were subsequently compared, and the corresponding polarization curves are shown in Fig. 4(B).

The OER electrocatalytic potential for different modified electrode materials was investigated with overpotentials(*η*_10_) at 10 mA cm^−2^ current density by using eqn (6):^75^

6 *η*_10_ = *E*_RHE_ − 1.23 V

According to the polarization data, the unmodified In-PGE electrode exhibited poor OER performance, requiring an overpotential of nearly 500 mV to attain a current density of 10 mA cm^−2^. In contrast, the Co_3_O_4_@Ac-PGE electrode displayed the most efficient catalytic behavior, achieving the same current density at only 240 mV. This pronounced improvement suggests that activation of the graphite surface, followed by Co_3_O_4_ deposition through SILAR process greatly enhanced the surface activity and charge transfer capability for OER. Intermediate OER activities were observed for Co_3_O_4_@In-PGE and Ac-PGE electrodes, which required overpotentials of approximately 280 mV and 440 mV, respectively, to deliver 10 mA cm^−2^ current density. The superior performance of Co_3_O_4_@Ac-PGE can be attributed to the synergistic effect between the activated carbon matrix and the homogeneously distributed Co_3_O_4_ particles, which collectively promote rapid electron transport and increase the density of exposed active sites.^76,77^ From Fig. 4B, the onset potentials for oxygen evolution were found to be around 1.43, 1.48, 1.51, and 1.73 V *vs.* RHE for Co_3_O_4_@Ac-PGE, Co_3_O_4_@In-PGE, Ac-PGE, and In-PGE, respectively. The notably lower onset and overpotential values observed for the Co_3_O_4_@Ac-PGE electrode demonstrate its excellent electrocatalytic efficiency and favorable charge transfer kinetics, indicating it as the most promising OER catalyst among the investigated modified electrodes.^78^ The LSV polarization curves presented in this work were corrected for the solution resistance (*iR* correction) using the uncompensated solution resistance (*R*_s_) obtained from electrochemical impedance spectroscopy (EIS). The corrected potential was calculated according to *E*_corrected_ = *E*_measured_ − *iR*_s_, where *i* is the measured current and *R*_s_ was determined from the high-frequency intercept of the Nyquist plot. To verify that the observed electrocatalytic performance was not influenced by the *iR* correction, the raw uncompensated LSV polarization curves and the corresponding Tafel plots are provided in the SI (Fig. S2). Furthermore, a comparison between the *iR*_s_-corrected and uncompensated electrochemical parameters (Table S3) reveals only slight increases in both overpotential and Tafel slope after removal of the *iR* correction. For the Co_3_O_4_@Ac-PGE electrode, the overpotential increased from 240 to 254.6 mV, while the corresponding Tafel slope increased from 47.6 to 62.3 mV dec^−1^. Similar minor differences were observed for the remaining electrodes. Importantly, the relative catalytic activity remained unchanged (Co_3_O_4_@Ac-PGE > Co_3_O_4_@In-PGE > Ac-PGE > In-PGE), confirming that the superior OER performance of the Co_3_O_4_@Ac-PGE electrode is intrinsic and not an artifact arising from the *iR* compensation. To evaluate the intrinsic electrocatalytic activity independent of the electrochemically active surface area, the polarization curves were further normalized by ECSA. As shown in Fig. S3, the ECSA-normalized polarization curves preserve the same activity trend observed in the geometric area-normalized measurements, confirming that the superior OER performance of the Co_3_O_4_@Ac-PGE electrode originates from its intrinsic catalytic activity rather than solely from an increased electrochemically active surface area.

Another important criterion for evaluating the kinetics of electrocatalysts is Tafel analysis, which makes it an essential parameter for examining the kinetics of the oxygen evolution reaction.^79^ In, Fig. 4C, polarization curves are fitted based on the Tafel eqn (7).7
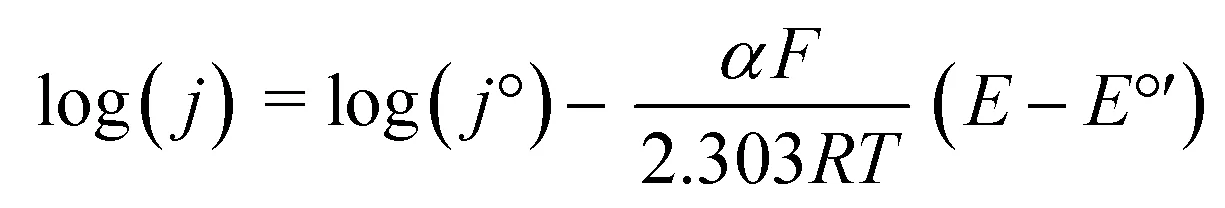
where, *E*°′, *E* and 
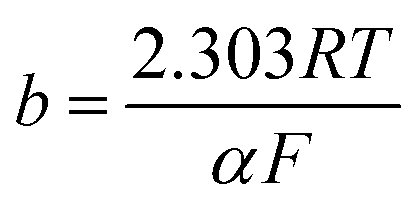
 refer to formal potential, applied potential (*vs.* RHE) and Tafel slope, respectively while the exchange current density is denoted as *j*_0_ = *nFCk*° at a state of *E* = *E*°′.

In general, a catalyst with a smaller Tafel slope value is more active since a lower overpotential is required to raise the current.^80^Table 1 represents the Tafel slope values for In-PGE, Ac-PGE, Co_3_O_4_@In-PGE, and Co_3_O_4_@Ac-PGE electrodes, respectively as 164.94, 152.88, 64, 64.91, and 47.57 mV/dec. Here, Co_3_O_4_@Ac-PGE electrode represents the lowest Tafel slope and the maximum value is observed at In-PGE. When we used the SILAR method to deposit Co_3_O_4_, the Tafel slope reduced, indicating improved OER kinetics when the electrode is modified by SILAR method. The smaller value of the Tafel slope for Co_3_O_4_@Ac-PGE suggests a faster kinetic rate for the catalyst, compared to that of the other three electrodes. All the data for the as-modified electrodes are summarized in Table 1.

**Table 1:** Onset potential, Tafel slope and Overpotential for OER at various electrodes in 1 M NaOH

Electrodes	Onset potential (V)	Tafel slope (mV dec^−1^)	Over potential (mV) at 10 mA cm^−2^
In-PGE	1.73	164.94	500
Ac-PGE	1.67	152.88	440
Co_3_O_4_@In-PGE	1.48	64.91	280
Co_3_O_4_@Ac-PGE	1.43	47.57	240

For the oxygen evolution process, pencil graphite-supported Co_3_O_4_ thin layer has a synergistic impact due to improved electron transport, increased surface area, and high conductivity, all of which work together to boost the performance of the electrocatalyst for OER.^81^

A major key factor in assessing OER kinetics is the turnover frequency (TOF). It is defined as the change of reactive molecules per unit site over a unit period of time.^82^ A higher TOF value indicates high efficiency of the catalytic material for a particular reaction.^83^ The TOF is typically estimated by two steps: (i) evaluating the catalytically active sites *m* and (ii) calculating the TOF value using eqn (8):^84^8
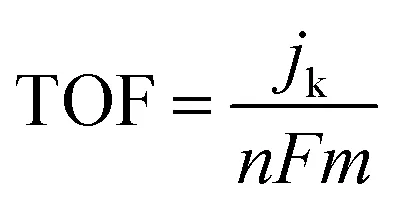
where, *j*_k_ (A cm^−2^) is the kinetic current density, *n* is the number of electrons transferred during the OER process, *F* = 96 485 C mol^−1^ is the Faraday constant, and *m* (mol cm^−2^) denotes the number of active sites.

Accurate determination of the electrochemically active surface area (ECSA) of the electrode for catalysis remains challenging. However, eqn (9) offers a convenient approach to estimate the double-layer capacitance (*C*_dl_) by correlating the capacitive current with the scan rate. The obtained *C*_dl_ value serves as a reliable proxy for the ECSA.^85^9
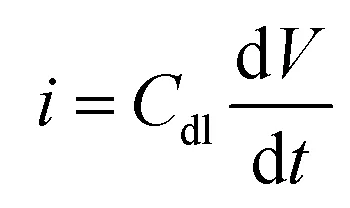


The *C*_dl_ values for In-PGE, Ac-PGE, Co_3_O_4_@In-PGE, and Co_3_O_4_@Ac-PGE electrodes were determined as 0.103, 0.118, 0.123 and 0.205 mF cm^−2^, respectively, based on the slope of the plot of current *vs.* scan rate in Fig. 4D. The corresponding CV curves recorded at various scan rates for *C*_dl_ calculations are presented in Fig. S4. The *C*_dl_ of the In-PGE electrode, which is taken as the bare electrode, is compared to the *C*_dl_ of the other electrodes, with the In-PGE surface area considered as the geometrical area. ECSA_modified_ was calculated using eqn (10) (ref. 86) and the comparative ESCAs were recorded in Table 3.10
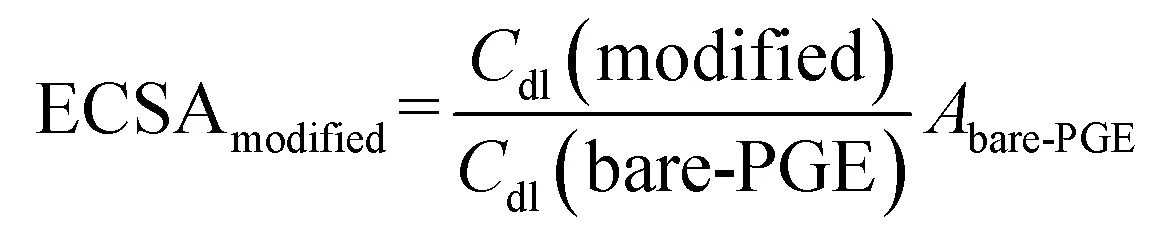


From the slope value of the current *vs.* scan rate plot (Fig. 6C) and the value of ECSA_modified_, the value of active site “*m*” was found using eqn (11) for different electrodes which are reported in Table 2.11
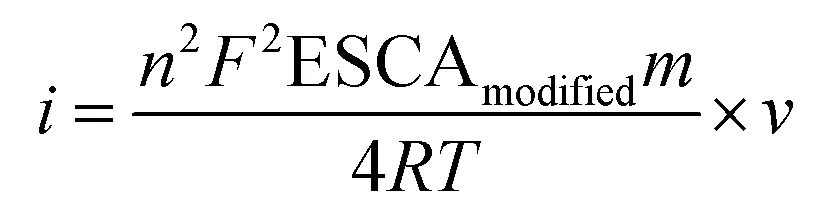


**Table 2:** The values for *C*_dl_, ECSA, *m* and TOF for OER at different electrodes

Electrodes	*C*_dl_ (mF cm^−2^)	ECSA (10^−3^ cm^2^)	*m* (10^−10^ mol cm^−2^)	TOF (s^−1^)
In-PGE	0.103	70	979.01	2.38 × 10^−3^
Ac-PGE	0.118	81	969.27	3.61 × 10^−3^
Co_3_O_4_@In-PGE	0.123	84.43	969.29	0.038
Co_3_O_4_@Ac-PGE	0.205	140.71	969.34	0.082

Consequently, the TOF value for OER was computed by using the value of ‘*m*’ derived from eqn (11) in eqn (8).^87^ The values are summarized in Table 2.

### 3.5 Stability of the catalyst

**Fig. 5:**
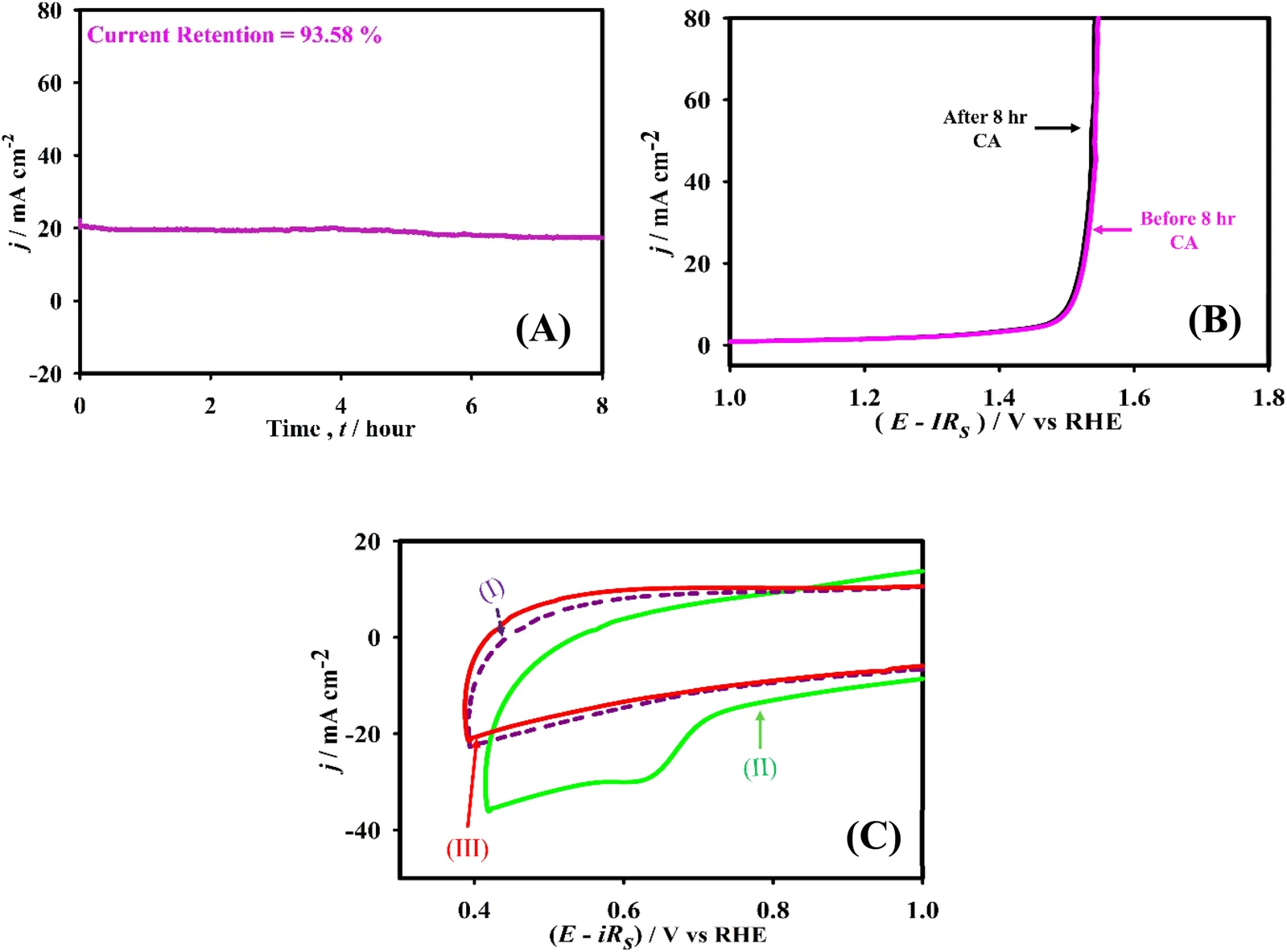
Stability test for Co_3_O_4_@Ac-PGE in 1.0 M NaOH (A) CA curve for Co_3_O_4_@Ac-PGE electrode at a constant potential of 1.55 V *vs.* RHE in 1.0 M NaOH solution, (B) LSV before and after 8 h Stability test(C) CV curves of Co_3_O_4_@Ac-PGE electrode: (I) before 8 h electrolysis by CA, (II) after 8 h electrolysis CA where significant amount of oxygen generated and the saturated in the electrolyte, and (III) in N_2_-saturated 1 M NaOH solution where dissolve oxygen is removed which were generated by 8 h CA stability test.

Based on the obtained results, the Co_3_O_4_@Ac-PGE electrode fabricated *via* the SILAR method demonstrated satisfactory electrochemical stability toward the oxygen evolution reaction (OER) in alkaline medium.^88,89^ The chronoamperometric (CA) response recorded at a constant potential of 1.55 V *vs.* RHE for 8 h in 1.0 M NaOH solution (Fig. 5A) showed only a gradual decrease in current density over time, where the electrode retained approximately 93.58% of its initial current density after prolonged operation. This relatively stable current response indicates good durability of the catalyst under continuous anodic polarization.^89–91^ Furthermore, the LSV curves recorded before and after the 8 h stability test (Fig. 5B) exhibited only a slight positive shift in potential with nearly preserved current density, suggesting that the catalytic activity of the electrode remained largely unaffected after long-term OER operation.^92,93^ The minimal variation in the polarization behavior implies that the catalyst maintains its electrochemically active surface during continuous electrolysis.^90,91^

A more detailed insight into the surface electrochemical behavior was obtained from the CV profiles shown in Fig. 5C. Notably, after the 8 h CA test, a distinct cathodic peak appeared around ∼0.62 V *vs.* RHE, which was absent before the stability measurement. This reduction peak response is likely associated with the electrochemical reduction of oxidized cobalt surface species formed during prolonged OER operation.^94,95^ Under anodic OER conditions, cobalt oxide-based electrocatalysts are known to undergo partial surface reconstruction, leading to the formation of higher-valence cobalt oxyhydroxide like species that act as catalytically active phases.^92,95,96^ Therefore, the appearance of the reduction peak after the CA test may indicate the reversible transformation of these surface-active cobalt species generated during oxygen evolution.

Importantly, the disappearance of the cathodic feature in the N_2_-saturated electrolyte after the CA measurement further suggests that the observed redox response is closely related to oxygen-associated surface intermediates formed during OER rather than irreversible structural degradation of the catalyst.^97,98^ This observation indicates that the electrochemical changes occurring at the catalyst surface are predominantly reversible in nature.^95,98^ Consequently, the retention of electrocatalytic activity together with the reversible redox behavior confirms good structural stability and reliable long-term OER performance of the Co_3_O_4_@Ac-PGE electrode in alkaline medium.^93,97^

**Fig. 6:**
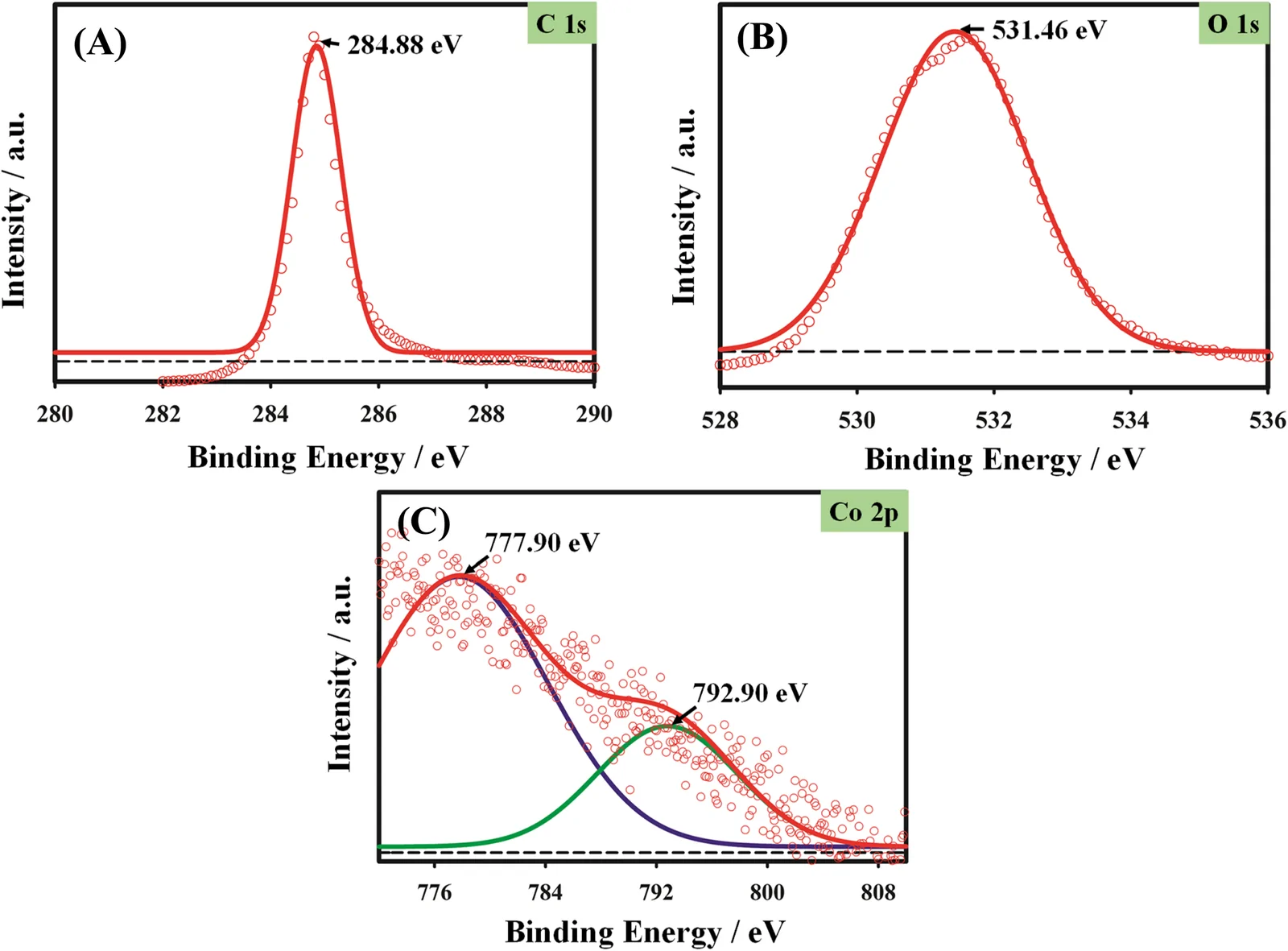
High-resolution post-electrochemical XPS spectra of the Co_3_O_4_@Ac-PGE electrode showing (A) C 1s, (B) O 1s, and (C) Co 2p regions.

To further elucidate the changes in surface chemistry and electronic structure induced by electrochemical operation, post-catalytic X-ray photoelectron spectroscopy (XPS) measurements were carried out on the cobalt oxide-functionalized electrode.^99^ The high-resolution spectra of C 1s, O 1s and Co 2p, presented in Fig. 6(A–C), provide valuable information regarding surface composition and the oxidation behavior of the catalyst after the electrochemical process.

The C 1s spectrum shown in Fig. 6A is dominated by a peak located at 284.88 eV, which corresponds to sp^2^ hybridized graphitic carbon originating from the graphite support, while relative to the before-catalysis electrode, a slight shift toward higher binding energy is observed, indicating modification of the electronic surroundings of the carbon matrix due to interaction with cobalt-containing species formed during operation. The strong intensity and well-defined nature of the graphitic carbon peak suggest that the conductive framework retains its structural integrity even after prolonged electrochemical use. Such preservation of the carbon network is essential for maintaining efficient electron transfer during the OER.^100,101^ The O 1s spectrum in Fig. 6B exhibits a broad feature centered at 531.46 eV, which can be assigned to oxygen-containing species such as surface hydroxyl groups and metal oxygen bonds. Compared with the spectrum before catalysis, the slight negative shift in binding energy reflects a change in oxygen coordination and stronger interaction between oxygen species and cobalt centers. The enhanced oxygen contribution further indicates increased surface hydroxylation and the probable generation of cobalt oxyhydroxide like phases during electrochemical activation. These oxygen rich surface species are known to facilitate adsorption of reaction intermediates and thereby accelerate OER kinetics.^102^

The Co 2p spectrum displayed in Fig. 6C contains two principal peaks located at 777.90 eV and 792.90 eV, corresponding to the Co 2p_3/2_ and Co 2p_1/2_ states, respectively. The broadened and asymmetric peak profiles indicate the coexistence of multiple cobalt oxidation states, primarily Co^2+^ and Co^3+^, characteristic of electrochemically transformed cobalt oxide or oxyhydroxide species. The variation in the cobalt electronic environment after catalysis suggests active surface transformation during OER operation. Such mixed-valence cobalt centers are highly beneficial for catalytic activity because they promote reversible redox transitions and improve interfacial charge-transfer processes.^103–105^

The post reaction XPS confirms the surface transformation into oxygen rich cobalt oxyhydroxide species that strengthened Co-carbon electronic interaction enhancing electron transfer, intermediate adsorption, stability and overall OER activity.

Finally, Table 3 summarizes the OER performance of representative Co_3_O_4_-based electrocatalysts fabricated using different synthesis and deposition strategies on various substrates in alkaline media. The comparison is based on key electrochemical parameters, including overpotential and Tafel slope, which are commonly used to evaluate OER activity and reaction kinetics.

**Table 3:** Comparison of OER catalytic performance of Co_3_O_4_-based electrodes in alkaline solution^a^

Catalysts	Medium	Method	Materials	Substrate	*η*_10_/mV	*b*/mV dec^−1^	Ref.
Co_3_O_4_/EG	1 M KOH	Hydrothermal	Co_3_O_4_ NPs	Graphite	301	47	106
Co_3_O_4_@g-C_3_N_4_	1 M KOH	Hydrothermal + calcination	Co_3_O_4_ nanoparticles	g-C_3_N_4_ nanosheets	340	120.92	107
Co_3_O_4_@C	1M KOH	Electrodeposition	Co_3_O_4_ nanoparticles	CC	370	107	108
Gr@eCo_3_O_4_	1 M KOH	Electrodeposition	Co_3_O_4_ + Gr + SiO_2_	GCE	330	45	109
Co_3_O_4_	1 M KOH	Commercial	Co_3_O_4_	GCE	405	75	109
Co_3_O_4_	1 M KOH	Annealing	Co_3_O_4_	GCE	420	55	110
Co_3_O_4_/NRGO	1 M KOH	Hydrothermal	rGO + Co_3_O_4_	GCE	370	80	111
Co_3_O_4_/NG	1 M KOH	Laser irradiation	NG + Co_3_O_4_	GCE	398	115	112
Co_3_O_4_/P–CN	1 M KOH	Hydrothermal	Co_3_O_4_ NPs	GCE	320	66.8	113
Co_3_O_4_@Ac-PGE	1 M NaOH	SILAR	Co_3_O_4_	PGE	240	47.57	This work

a NB. *η*_10_: overpotential at 10 mA cm^−2^, *b*: Tafel slope, NRGO: N-doped reduced graphene oxide, NG: N-doped graphene, N-gas: N-doped graphene aerogels, GCE: graphite carbon electrode CC: carbon cloth, PGE: pencil graphite electrode, SILAR: successive ionic layer adsorption and reaction.

As shown in Table 3, the OER performance of Co_3_O_4_-based electrocatalysts varies considerably depending on the synthesis route, deposition strategy, and substrate employed. The Co_3_O_4_@Ac-PGE electrode fabricated *via* the SILAR method exhibits competitive OER activity in comparison with previously reported Co_3_O_4_-based systems, indicating efficient catalyst integration onto the activated pencil graphite substrate. This improved performance is likely associated with the controlled layer by layer deposition afforded by the SILAR process, which facilitates effective utilization of active sites. For comparative evaluation, Co_3_O_4_ was also deposited *via* the drop-casting method, and the detailed analysis is provided in the Fig. S5 (SI), further highlighting the advantage of the SILAR approach in achieving enhanced overall electrochemical behavior.

Overall, this study demonstrates a robust and scalable strategy for the rational design of Co_3_O_4_@Ac-PGE, offering strong potential for advanced energy conversion and sustainable water oxidation applications.

## 4. Conclusion

This study delivers a simple, affordable, and scalable approach to developing efficient electrocatalysts for energy conversion. Turnover frequency and active site analysis verified high intrinsic activity, a favorable Tafel slope, and a low overpotential for the SILAR-deposited and electrically activated Co_3_O_4_@Ac-PGE electrode, exhibiting high catalytic performance. Chronoamperometric analysis further demonstrated the system's exceptional long-term stability with low current loss. In addition to its outstanding performance, Co_3_O_4_@Ac-PGE could be a desirable candidate for real world use in renewable energy technologies due to its simple and affordable fabrication process. Significantly, the knowledge gained from this work provides a strong basis for improving electrocatalyst stability, scalability, and efficiency even more. This opens the door to large-scale, sustainable energy conversion systems that can support global initiatives for the development of clean energy resulting in environmental protection.

## Conflicts of interest

The authors declare no conflict of interest.

## Funding

This work was supported by the Deanship of Scientific Research, Vice Presidency for Graduate Studies and Scientific Research, King Faisal University, Saudi Arabia [Grant No. KFU263662]. The authors also would like to thank Research Center of Shahjalal University of Science and Technology, Bangladesh for another grant (No. PS/2025/1/03).

## Supplementary Material

RA-016-D6RA04902H-s001

## Data Availability

The data supporting the findings of this study are provided within the article and its supplementary information (SI). Further information and supporting data are available from the corresponding author upon reasonable request. Supplementary information: Fig. S1: polarization curves of different as-modified electrodes in alkaline medium, Fig. S2: raw uncompensated LSV polarization curves for optimized Co_3_O_4_ deposition through different SILAR cycles, corresponding Tafel plots, Fig. S3: ECSA-normalized polarization curves through variation of SILAR cycles (1–30) and at a scan rate of 10 mV s^−1^, Fig. S4: CV curves obtained at different scan rates for *C*_dl_ calculation for different electrodes, Fig. S5: comparison of SILAR with other methods such as drop casting, comparison of stability and comparative LSV curves recorded before and after 8 h CA test in 1 M NaOH solution, Fig. S6: polarization curves replotted as potential (*E*) *versus* current density (*j*) for different electrodes, Table S1: EIS parameters of different electrodes in 1 M NaOH solution, Table S2: comparative electrocatalytic OER performance of Co_3_O_4_-modified PGE prepared by varying SILAR deposition cycles (1–30) in 1.0 M NaOH, Table S3: comparison of *iR*_s_-corrected and uncompensated electrochemical parameters. See DOI: https://doi.org/10.1039/d6ra04902h.
