# Supplementary material for: Successive ionic layer adsorption and reaction (SILAR) -driven cobalt oxide integration on pencil graphite for efficient electrochemical oxygen evolution reaction in alkaline medium
Source: RSC Adv. 2026 Jul 29. Online ahead of print. doi: 10.1039/d6ra04902h (PMC13418240; doi:10.1039/d6ra04902h)
Supplement: RA-OLF-D6RA04902H-s001 [file RA-OLF-D6RA04902H-s001.pdf]

Supplementary Information (SI) for RSC Advances.
This journal is © The Royal Society of Chemistry 2026

Supporting Information

Successive Ionic Layer Adsorption and Reaction -Driven Cobalt Oxide Integration on Pencil Graphite for Efficient Electrochemical Oxygen Evolution Reaction in Alkaline Medium.

Mahfuz Rana [a], Kazi Hamidur Rashid [b], Abrar Yasir Abir [a], Faiza Jan Iftikhar [c], Motasim Bin Islam [b], Md Mahmudul Hasan [d], Mohammad Anwar Parvez [e], Mostafizur Rahaman [f], Syed Kashif Ali [g], Mohammad A. Hasnat [b, *]

[a] *Department of Chemistry, Pabna University of Science and Technology, Pabna-6600, Bangladesh*

[b] *Electrochemistry & Catalysis Research Laboratory (ECRL), Department of Chemistry, School of Physical Sciences, Shahjalal University of Science and Technology, Sylhet-3114, Bangladesh*

[c] *NUTECH School of Applied Sciences & Humanities, National University of Technology, Islamabad 44000, Pakistan*

[d] Research Organization for Nano & Life Innovation, Waseda University, Japan

[e] *Department of Chemical Engineering, College of Engineering, King Faisal University, P.O. Box 380, Al-Ahsa 31982, Saudi Arabia*

[f] *Department of Chemistry, College of Science, King Saud University, P.O. Box 2455, Riyadh 11451, Saudi Arabia*

[g] *Department of Physical Sciences, Chemistry Division, College of Science, Jazan University, P.O. Box. 114, Jazan 45142, Kingdom of Saudi Arabia*

Corresponding author: Mohammad A. Hasnat

E-mail address: mah-che@sust.edu

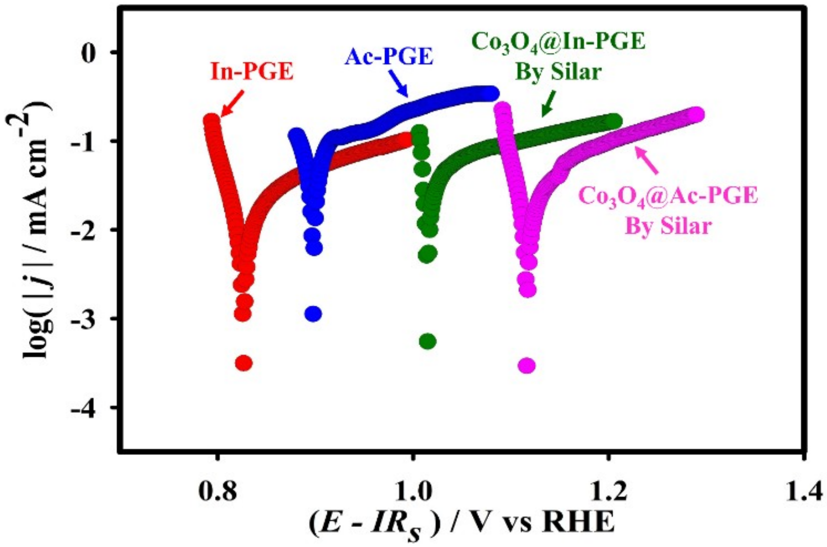

Figure S1. Polarization curves for In-PGE, Ac-PGE, Co_3O_4@In-PGE, Co_3O_4@Ac-PGE in alkaline medium.

Table S1. EIS parameters of different electrodes recorded at a potential of 1.55 V vs. RHE in 1 M NaOH solution.

Electrodes	$R_s(\Omega)$	$R_{ct}(k\Omega)$
**In-PGE**	25.7	3.50
**Ac-PGE**	30.3	2.29
**Co_3O_4@In-PGE**	29.1	0.12
**Co_3O_4@Ac-PGE**	33.1	0.08

Table S2. Comparative electrocatalytic OER performance of Co_3O_4-modified PGE prepared by varying SILAR deposition cycles (1–30) in 1.0 M NaOH.

SILAR cycles	Onset potential (V vs. RHE)	Overpotential at 10 mA cm^{-2} (mV)	Tafel slope (mV dec^{-1})
1	1.5	288.3	60.71
3	1.51	286	52.5

5	1.43	240	47.57
10	1.48	272.1	54.08
20	1.49	273	50.3
30	1.47	269	56.29

The electrochemical parameters were extracted from linear sweep voltammetry (LSV) measurements in 1.0 M NaOH. Among all prepared electrodes, the sample synthesized with 5 SILAR deposition cycles exhibited the most favorable OER activity and was therefore selected for further investigations.

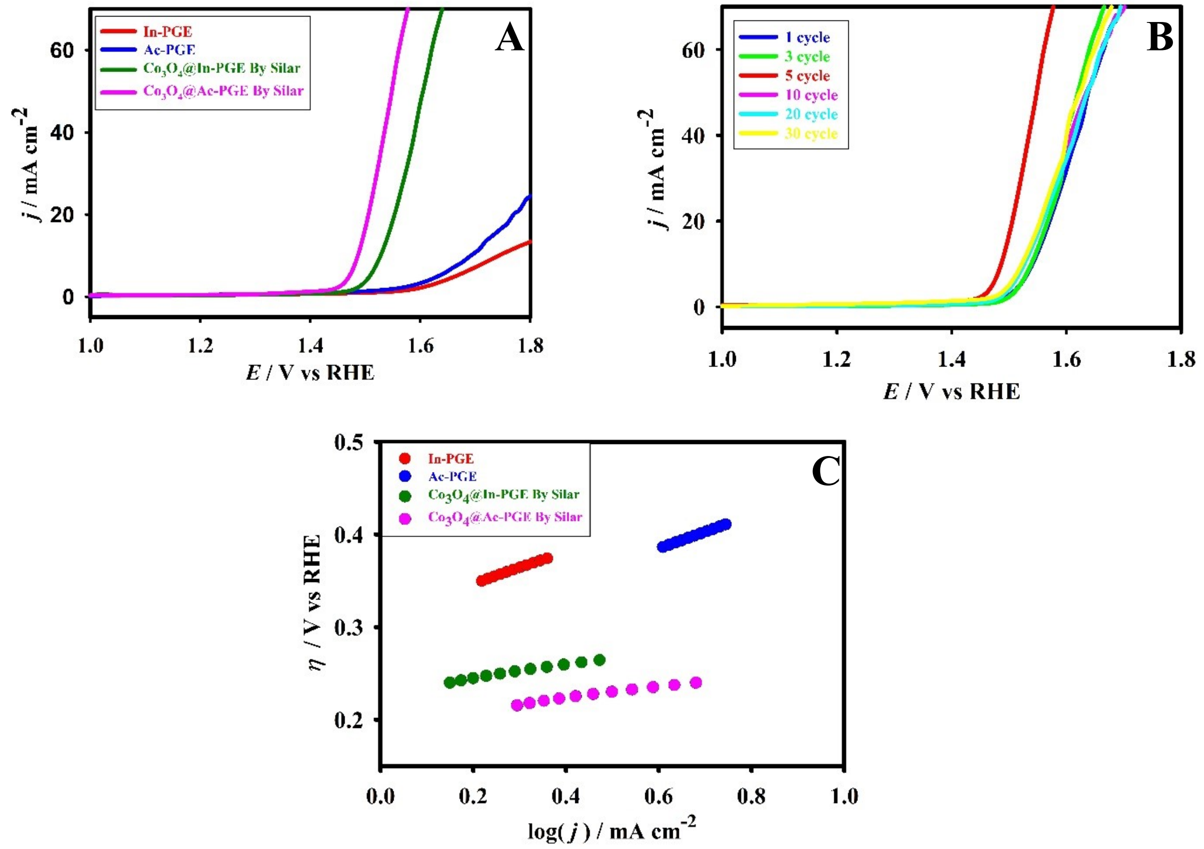

Figure S2. (A) Raw uncompensated LSV polarization curves of In-PGE, Ac-PGE, Co_3O_4@In-PGE, and Co_3O_4@Ac-PGE. **(B)** Raw uncompensated LSV polarization curves for the optimization of Co_3O_4 deposition through different SILAR cycles (1–30). **(C)** Corresponding Tafel plots derived from the raw uncompensated LSV polarization curves.

Table S3: Comparison of iR_s-corrected and uncompensated electrochemical parameters.

Electrode	Overpotential at 10 mA cm^{-2} (mV) (iR_s-corrected)	Overpotential at 10 mA cm^{-2} (mV) (Uncompensated)	Tafel slope (iR_s-corrected) (mV dec^{-1})	Tafel slope (Uncompensated) (mV dec^{-1})
In-PGE	500	510.9	164.9	172.9
Ac-PGE	440	459.7	152.9	180.1
Co_3O_4@In-PGE	280	296.1	64.9	74.8
Co_3O_4@Ac-PGE	240	254.6	47.6	62.3

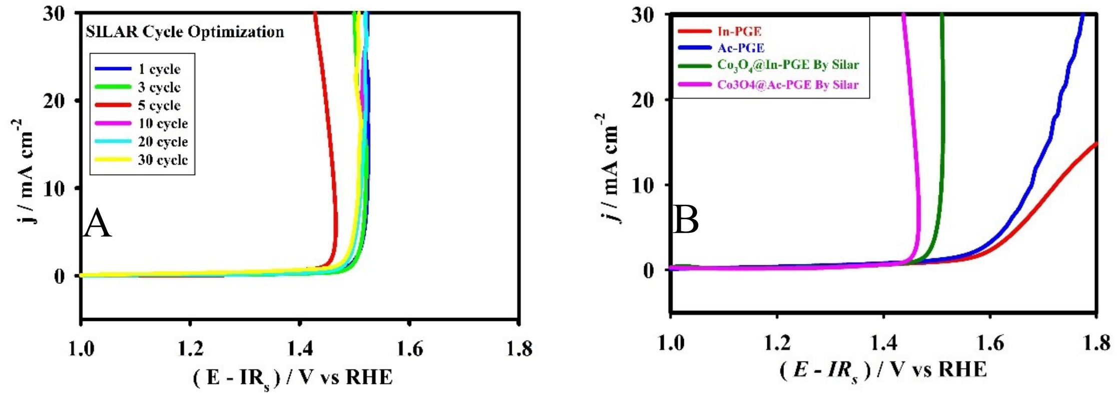

Figure S3: (A) ECSA-normalized polarization curves showing the optimization of Co_3O_4 deposition through variation of SILAR cycles (1–30). **(B)** ECSA-normalized polarization curves of In-PGE, Ac-PGE, Co_3O_4@In-PGE, and Co_3O_4@Ac-PGE electrodes recorded at a scan rate of 10 mV s^{-1}.

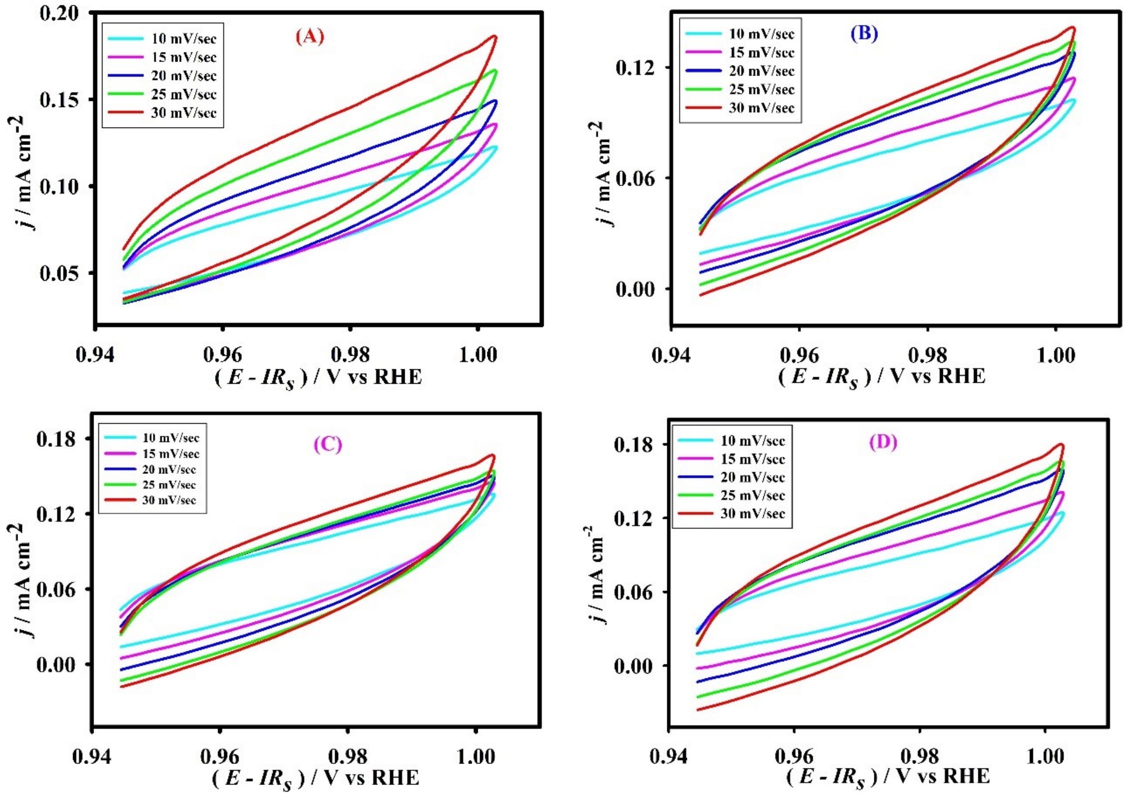

Figure S4. CV curves obtained at different scan rates for the estimation of C_{dl} values by plotting current density versus scan rate for (A) In-PGE, (B) Ac-PGE, (C) Co_3O_4@In-PGE, and (D) Co_3O_4@Ac-PGE electrodes.

Comparison with other methods

Here, the SILAR method is compared with the other established methods to analyze the opportunity for practical applicability of the developed electrocatalyst for OER. For this, the Co_3O_4/PGE electrode is prepared by the traditional drop-casting method, and the electrochemical responses, including LSV and chronoamperometric (CA) stability, are presented in **Figure S5(A-C)**. **Figure S5A** shows the LSV profile of the drop-casted electrode, while **Figure S5B** presents the CA stability response over 8 h. The corresponding LSV curves recorded before and after the CA test are shown in **Figure S5C.**

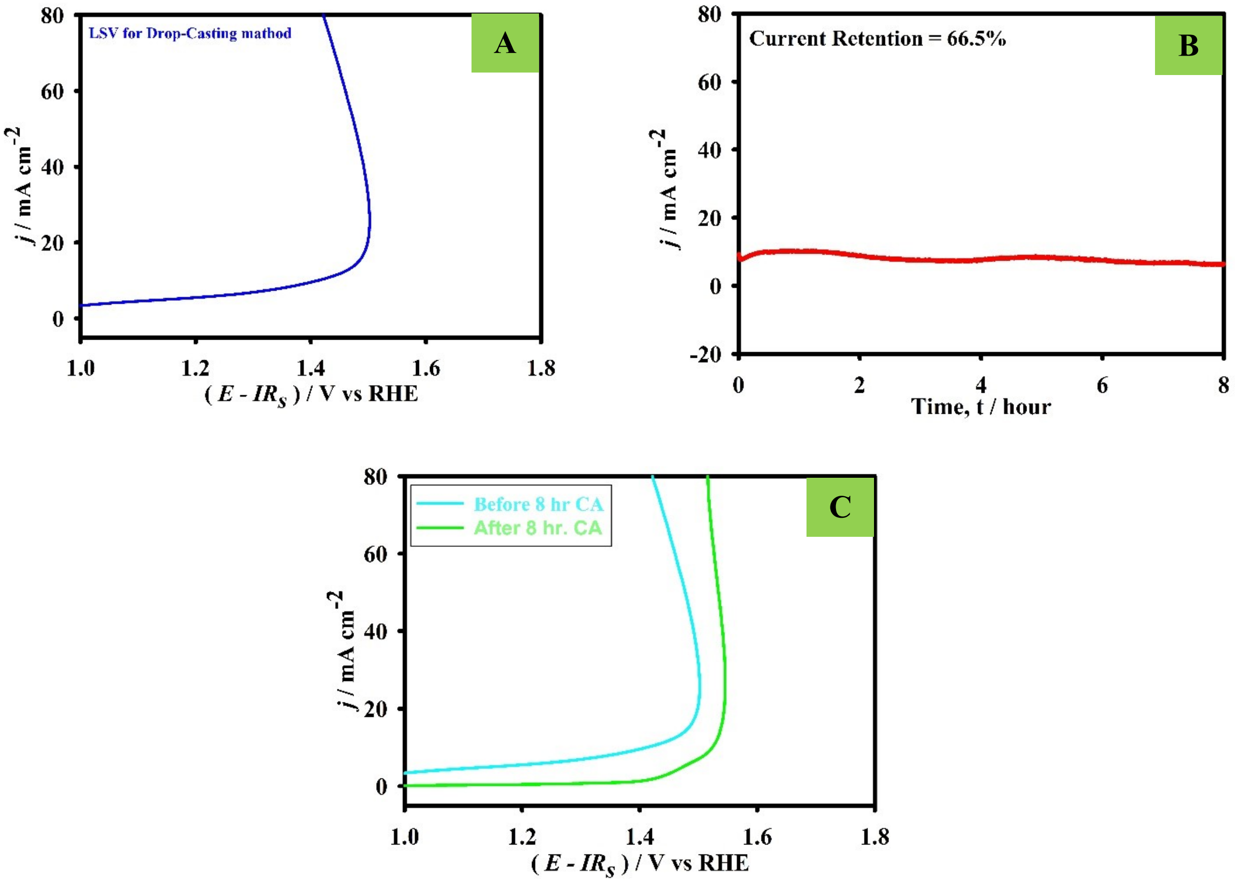

Figure S5. (A) LSV curve of the drop-casted modified PGE, (B) chronoamperometric (CA) response demonstrating the stability of the electrode over 8 h, and (C) comparative LSV curves recorded before and after 8 h CA test in 1 M NaOH solution.

At a current density of 10 mA cm^{-2}, the drop-casted electrode exhibits a slightly lower overpotential (236.5 mV) compared to the SILAR-derived electrode (240 mV). However, the

overall electrochemical behavior suggests that the SILAR method provides superior catalytic performance in terms of kinetics and long-term stability. It is important to emphasize that although both electrodes were fabricated using the same Co_3O_4 material on identical PGE substrates and evaluated under identical experimental conditions (1 M NaOH electrolyte and identical electrochemical protocols), the fabrication method significantly influenced their electrocatalytic performance [1,2].

This is further supported by the onset potential values, where the SILAR electrode (1.43 V) shows a lower value than the drop cast electrode (1.47 V), indicating earlier initiation of oxygen evolution and improved intrinsic catalytic activity [3]. Kinetic analysis reveals a clear distinction between the two methods. The SILAR electrode shows a lower Tafel slope (47.57 mV dec^{-1}) compared to the drop-casted electrode (53.3 mV dec^{-1}), confirming faster charge-transfer kinetics and more efficient reaction pathways [4].

Stability results also strongly favor the SILAR method. After 8 h of chronoamperometric test, the SILAR electrode retains 93.58% of its initial current, whereas the drop casting electrode retains only 66.5%, demonstrating better durability and structural stability [5]. Moreover, the pre and post-stability LSV curves of the SILAR electrode nearly overlap, indicating excellent electrochemical stability with negligible degradation [6]. In contrast, the drop casting electrode shows a noticeable deviation after stability testing, suggesting partial loss of active material or surface restructuring during operation [7].

This difference can be attributed solely to the fabrication method. The drop casting approach uses Nafion binder for catalyst adhesion, which partially blocks active sites and increases interfacial resistance [8]. In contrast, the SILAR method produces a binder-free Co_3O_4 coating directly on the electrode surface, ensuring improved electrical contact, efficient charge transfer, and higher accessibility of active sites [9].

Therefore, despite both electrodes having identical chemical composition and being tested under the same experimental conditions, the fabrication strategy plays a crucial role in determining their OER activity. The SILAR-derived Co_3O_4 electrode demonstrates superior electrocatalytic performance through lower onset potential, improved reaction kinetics, enhanced current retention, and remarkable electrochemical stability. These results confirm that the binder-free and directly grown Co_3O_4 architecture produced by the SILAR method provides more efficient charge transfer and greater accessibility of active sites, making it a more effective and durable approach for developing high-performance OER electrocatalysts.

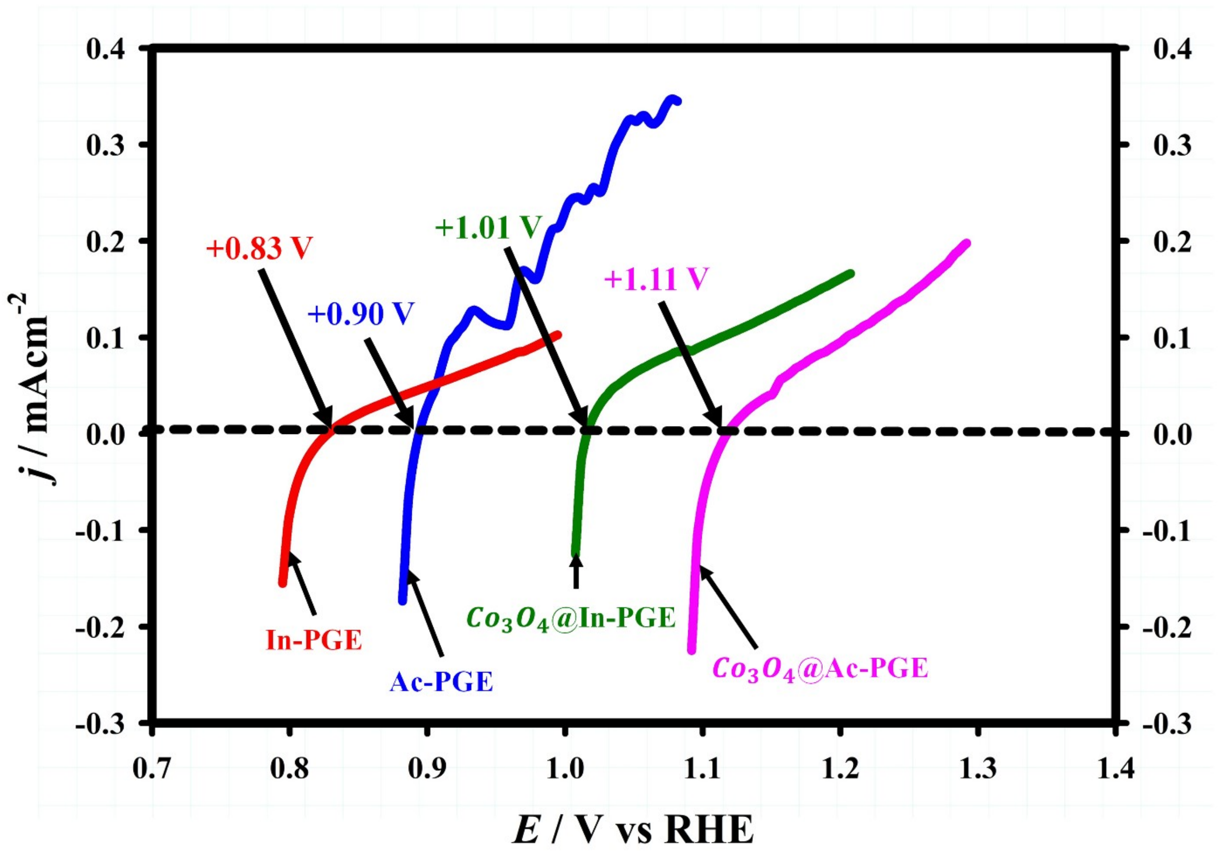

Figure S6. Polarization curves replotted as potential (E) versus current density (j) for In-PGE, Ac-PGE, Co_3O_4@In-PGE, and Co_3O_4@Ac-PGE, provided in response to the reviewer's comment. The arrows indicate the OER onset potentials.

References

1. Kale, M. B., Borse, R. A., Gomaa Abdelkader Mohamed, A. & Wang, Y. Electrocatalysts by Electrodeposition: Recent Advances, Synthesis Methods, and Applications in Energy Conversion. *Adv. Funct. Mater.* **31**, 2101313 (2021).

2. Li, D. C., Muta, T., Zhang, L. Q., Yoshio, M. & Noguchi, H. Effect of synthesis method on the electrochemical performance of LiNi1/3Mn1/3Co1/3O2. *J. Power Sources* **132**, 150–155 (2004).

3. Ede, S. R. & Luo, Z. Tuning the intrinsic catalytic activities of oxygen-evolution catalysts by doping: a comprehensive review. *J. Mater. Chem. A Mater.* **9**, 20131–20163 (2021).

4. García-Osorio, A. *et al.* The Kinetic Parameters of the Oxygen Evolution Reaction (OER) Calculated on Inactive Anodes via EIS Transfer Functions: •OH Formation. *J. Electrochem. Soc.* **164**, E3321 (2017).

5. Khan, I. A., Morgen, P., Sharma, R. & Andersen, S. M. Limitations of Chronopotentiometry Test Protocols for Stability Study on Oxygen Evolution Reaction Electrocatalysts and Recommendations. *The Journal of Physical Chemistry C* **128**, 2828–2833 (2024).

6. Jamadar, A. S., Sutar, R. B., Deshpande, N. G., Salunkhe, R. R. & Yadav, J. B. Highly durable, Pt free and large-area Ni–B film synthesized by SILAR as a bifunctional electrode for electrochemical water splitting. *Int. J. Hydrogen Energy* **47**, 34492–34503 (2022).

7. Drop-cast gold nanoparticles are not always electrocatalytically active for the borohydride oxidation reaction - Chemical Science (RSC Publishing) DOI:10.1039/D4SC00676C. https://pubs.rsc.org/en/content/articlehtml/2024/sc/d4sc00676c.

8. Lin, Y., Shi, K., Yang, Y., Yang, Z. & Zhang, W. Polydopamine-engineered design of Nafion-free powder electrode with superhydrophilicity/superaerophobicity and superior adhesive structure for efficient overall water splitting. *Chem. Eng. Sci.* **281**, 119125 (2023).

9. Patil, V. V. *et al.* SILAR Synthesized Binder-Free, Hydrous Cobalt Phosphate Thin Film Electrocatalysts for OER Application: Annealing Effect on the Electrocatalytic Activity. *Int. J. Energy Res.* **2023**, 5570480 (2023).
